# DQAgui: a graphical user interface for the MIRACUM data quality assessment tool

**DOI:** 10.1186/s12911-022-01961-z

**Published:** 2022-08-11

**Authors:** Jonathan M. Mang, Susanne A. Seuchter, Christian Gulden, Stefanie Schild, Detlef Kraska, Hans-Ulrich Prokosch, Lorenz A. Kapsner

**Affiliations:** 1 Medical Center for Information and Communication Technology, Universitätsklinikum Erlangen, Erlangen, Germany; 2 Chair of Medical Informatics, Friedrich-Alexander-Universität Erlangen-Nürnberg (FAU), Erlangen, Germany; 3 Institute of Radiology, Universitätsklinikum Erlangen, Friedrich-Alexander-Universität Erlangen-Nürnberg, Erlangen, Germany

**Keywords:** Data quality assessment (DQA), Data accuracy, Electronic health records (EHR), Feasibility studies, Mobile applications, Documentation, User–computer interface

## Abstract

**Background:**

With the growing impact of observational research studies, there is also a growing focus on data quality (DQ). As opposed to experimental study designs, observational research studies are performed using data mostly collected in a non-research context (secondary use). Depending on the number of data elements to be analyzed, DQ reports of data stored within research networks can grow very large. They might be cumbersome to read and important information could be overseen quickly. To address this issue, a DQ assessment (DQA) tool with a graphical user interface (GUI) was developed and provided as a web application.

**Methods:**

The aim was to provide an easy-to-use interface for users without prior programming knowledge to carry out DQ checks and to present the results in a clearly structured way. This interface serves as a starting point for a more detailed investigation of possible DQ irregularities. A user-centered development process ensured the practical feasibility of the interactive GUI. The interface was implemented in the R programming language and aligned to Kahn et al.’s DQ categories conformance, completeness and plausibility.

**Results:**

With DQAgui, an R package with a web-app frontend for DQ assessment was developed. The GUI allows users to perform DQ analyses of tabular data sets and to systematically evaluate the results. During the development of the GUI, additional features were implemented, such as analyzing a subset of the data by defining time periods and restricting the analyses to certain data elements.

**Conclusions:**

As part of the MIRACUM project, DQAgui is now being used at ten German university hospitals for DQ assessment and to provide a central overview of the availability of important data elements in a datamap over 2 years. Future development efforts should focus on design optimization and include a usability evaluation.

**Supplementary Information:**

The online version contains supplementary material available at 10.1186/s12911-022-01961-z.

## Background

With the growing impact of observational research studies driven by digitalization processes and the establishment of large multicenter research networks [1, 2], which benefit from the increasing availability of electronic health records (EHR) [3–5], there is also a growing focus on data quality (DQ) [6, 7]. As opposed to experimental study designs where DQ is ensured by good clinical practice guidelines (GCP), observational research studies are conducted by using data that is primarily collected in a non-research context, such as documentation or billing purposes [7–9], also called “secondary use”. The assessment of DQ in this context and in such multisite research networks, however, comprises some challenges and several methods and approaches have been suggested to address them [7, 10, 11]. A goal of MIRACUM (Medical Informatics in Research and Care in University Medicine) [12], a data sharing network to enhance translational research and medical care funded by the German Medical Informatics Initiative (MII) [13], is to establish a privacy-preserving infrastructure that provides clinical routine data from electronic health records in research data repositories to be used in cross-institutional observational health studies. To provide such an infrastructure, all participating German university hospitals within the MII established local *Data Integration Centers* (DIC). The MII ensures a nationwide and inter-consortial interoperability by using the data sharing standard HL7® FHIR®. Besides that, each MIRACUM site integrates its routine data in a project-wide harmonized manner within the two data models *i2b2* [14] and *OMOP CDM* [15].

In order to be able to use EHR data to answer research questions, they should be checked for quality, including conformance (“Do data values adhere to specified standards and formats?”), completeness (“Are data values present?”), and plausibility (“Are data values believable?”) [7, 16–18]. For large data sets such as EHR, manual review is not feasible and automated processes should be used [16].

One principle of the MIRACUM project was to provide its concepts and implementations open source. Since an open source software tool addressing the need to ensure DQ across the research repositories with heterogeneous data models was lacking at the beginning of the project, the authors developed a *DQ Assessment* (DQA) tool and they demonstrated its connection to the *MIRACUM MDR* (M-MDR), which allows centralized management of all data elements and required DQ definitions and ensures that DQ checks are performed at each site in a standardized manner [19, 20].

Currently, the DQA tool consists of the R package DQAstats and provides a PDF report that includes the DQ check results of either a single database or two databases being compared. To create this PDF report, users currently need to configure the tool and execute commands in the R console, thus requiring some R programming knowledge as a prerequisite to use the tool.

To eliminate this requirement, a GUI was considered to be straightforward to use even for users without a technical background, for example via a web-browser.

The goal was to enable a broader range of users to use the DQA tool and perform DQ analyses. The GUI should be developed in an iterative, user-centered manner to ensure end-user acceptance. Although being driven by the MIRACUM projects use case of analyzing health data, the DQA tool was intended to be context independent and applicable to other (tabular) data sets.

## Methods

### Data quality framework

The lack of standardized terminology regarding DQ aspects in the literature led Kahn et al. in 2016 to initiate a harmonized three-category framework stating that each of these categories “conformance”, “completeness”, and “plausibility” can be interpreted in the two contexts of “verification” and “validation” [7]. Since this framework is well established and used in a large number of scientific papers [16, 21–24], the development of the MIRACUM DQ software is also aligned with it. Details on the specific implementation of the various DQ categories in DQAstats are described in a previous publication of the authors [20].

### Software

Static PDFs generated by DQAstats help to create verifiable reports, such as on the status of the DQ of a data set. However, depending on the number of data elements analyzed, the report can grow very large, making it quite cumbersome to read. Moreover, important information might be overseen. The GUI should address these points and include all the information in the PDF, but present it in a structured and clear manner. Users should be able to navigate through the GUI and explore the results intuitively. They should also be able to connect to databases for DQ analyses without technical knowledge. Since DQAstats always analyzes all available data elements, the GUI should provide a way to limit the amount of analyzed data to improve performance.

**Fig. 1:**
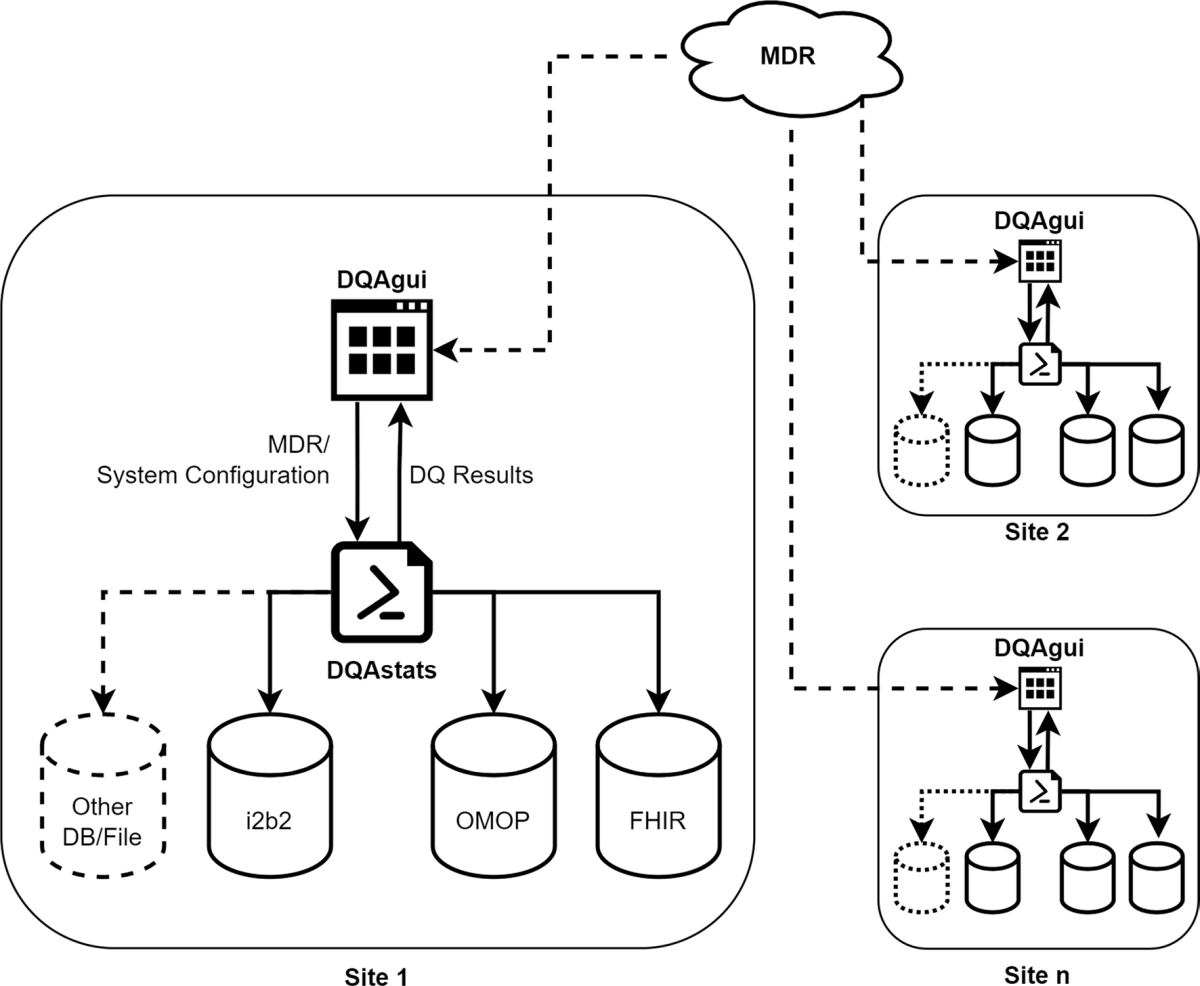
Integration of DQAgui into the data integration center (DIC) environment (schema). DQAgui directly builds upon DQAstats to serve as an interface for configuring the connection to the databases, carrying out the data quality (DQ) analyses, and visualizing the results via a web-based user interface. Within MIRACUM, the metadata repository (MDR) provides a centralized management of all data elements and required DQ definitions, and ensures that DQ checks are performed in a standardized manner across multiple sites using the same up-to-date criteria

To address the previously mentioned points, a GUI was developed to provide a frontend to the functions from the R package DQAstats [20]. The GUI itself is provided with the new R package DQAgui and directly builds upon DQAstats to serve as an interface to configure the connection to databases, carry out DQ analyses, and visualize the results via a web-based user interface (see Fig. 1).

**Fig. 2:**
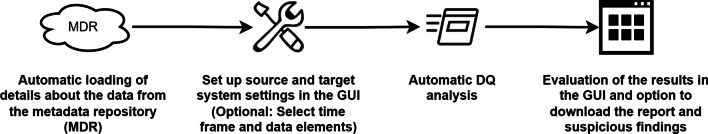
Schematic representation of the data quality (DQ) check process

The GUI was developed in the R programming language to allow a seamless integration of the DQAstats backend [25–27]. Figure 2 illustrates the workflow of a DQ check.

### User-centered development process

During the interface design, it was assumed that users were likely to be data stewards or data engineers with a limited background in R programming but at least some experience in the area of DQ. Based on these assumptions, technical prerequisites for using the GUI were minimized. As a first abstraction step to transition from a static PDF report to an interactive application, a working GUI prototype was developed to present the identical content of the previous report in a more structured and simplified manner. This first version of the DQA tool GUI was made available to all MIRACUM partner sites in September 2019. According to the MIRACUM project plan, all sites had to locally deploy the DQA tool and review their data collections for data quality. Simultaneously, several feedback channels were created, including a separate chat group. The channels provided the possibility to create issues in the source code repository and set up feedback pages to communicate problems or suggestions for improvement to the developers. According to the ergonomic principles defined in ISO 9241-110 [28], all findings were categorized and prioritized by their presumed severity and frequency (see Additional file 1: section “Feedback round 1 (FR1)” and Additional file 1: Fig. S1).

The collected user feedback from the first feedback phase, completed at the end of 2019, was incorporated into several software patches, allowing an intermittent minor release rolled out to all sites in April 2020. Further improvements were continuously implemented and published in an update of the DQA tool, which was made available to all partner sites in July 2021 and subsequently tested in the second feedback phase.

## Results

### Mapping command line functions to GUI elements

The sections from the PDF report created by DQAstats were gradually migrated to the GUI and tailored to the web interface. The summary overview of the completeness and conformance checks was integrated into the main GUI dashboard, which is automatically displayed after the completion of DQ analyses. The results of the automated comparison between two databases (completeness checks) are highlighted in color to attract attention to inconsistencies in the case of detected irregularities (see Additional file 1: Figs. S2 and S3). Tabs were introduced for easy navigation between the different DQ check results.

**Fig. 3:**
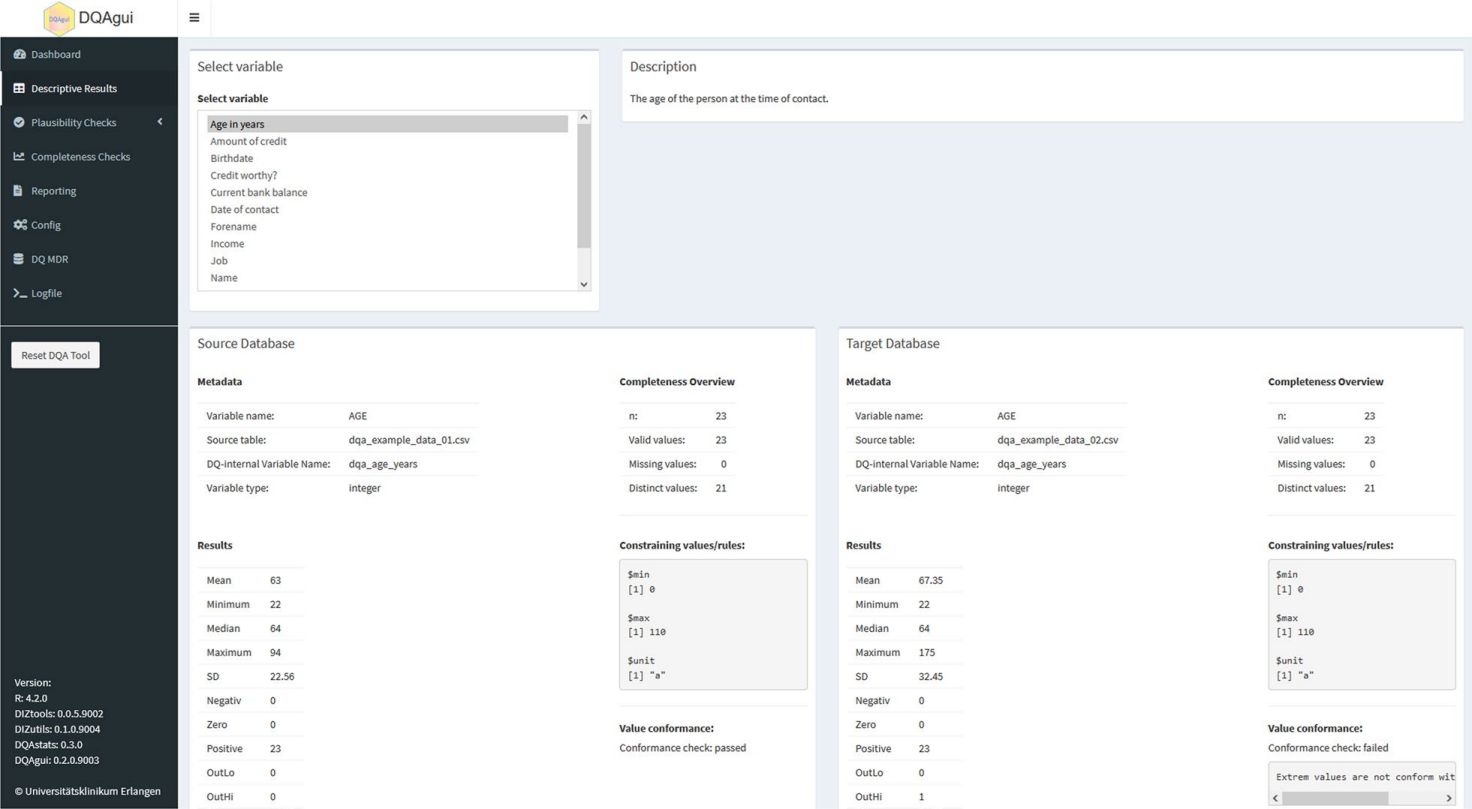
Representation of the descriptive analysis results for a single data element in the web-based interface. The results of the analysis of the selected data item are displayed on the left side for the source database and the right side for the target database

**Fig. 4:**
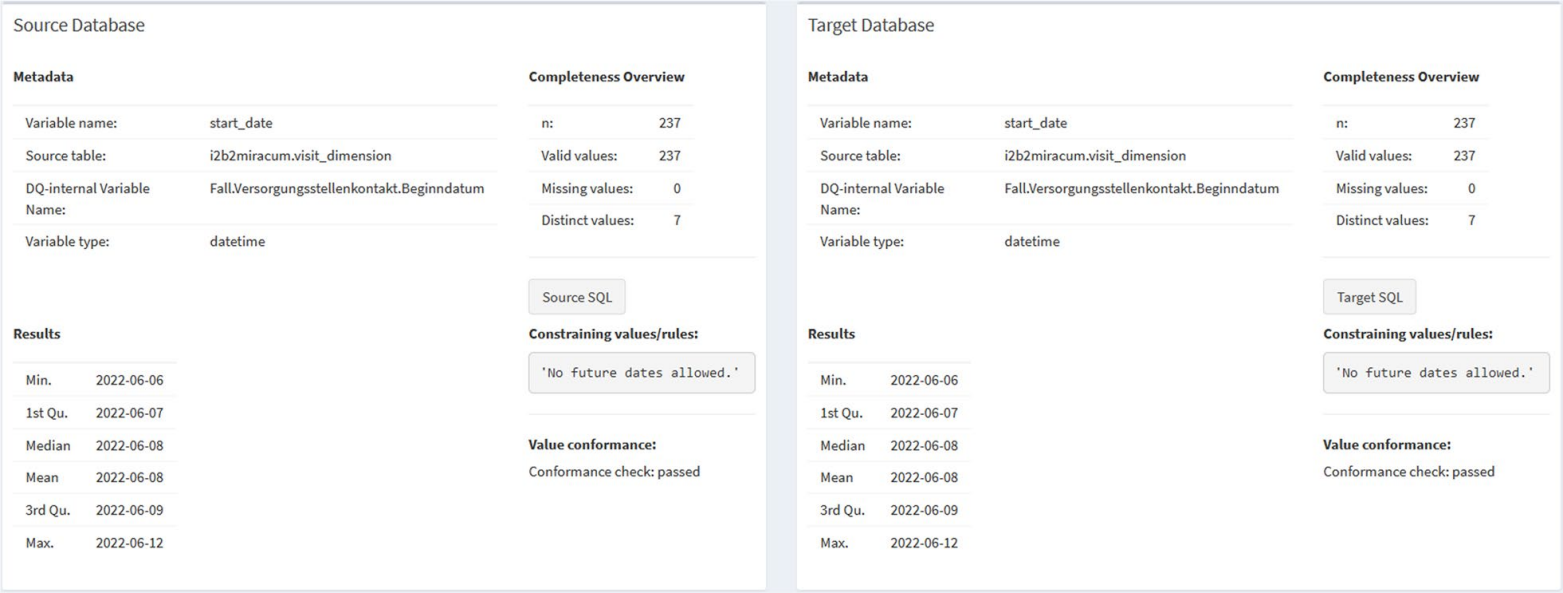
Summary screen for the descriptive analysis. For each data element in the descriptive analysis, results are enhanced with the ability to display the underlying SQL statement by the click of a button in order to quickly follow up on detected irregularities or data conformance violations in the source system by copying-and-pasting the SQL to a suited database management system

Characteristic details of each analyzed data element were previously provided in the PDF report’s section “Detailed Descriptive Results”. This information is now provided as a new GUI screen named *Descriptive Results*. Since the results of one data element are often dispersed throughout several pages in the PDF report (see Additional file 1: Fig. S4), comparing the characteristics of the data and the results of the DQ analysis in both systems can be cumbersome and error-prone. In the GUI, the findings are now displayed side by side for each data element in the source and target database to address this aspect, simplifying the comprehension of the results and enhancing direct comparison (see Figs. 3 and 4).

Similar to the PDF report, the adherence of a data element’s values to conformance criteria (value conformance) specified in the metadata repository (MDR) is presented in the descriptive results. Furthermore, the available metadata of the data element itself, such as the variable name, a short description, and its data type, as well as information on the data element’s mappings in the data sets, are also displayed here. For example, the table name from which the data element was loaded and the variable name in the respective database is visible. The visualization of the results of the DQ analyses depends on the variable type: basic distribution parameters (such as minimum, median, mean, standard deviation, and maximum) are calculated and displayed for numeric values or dates, whereas unique values and frequency counts are shown for categorical data elements or strings (see Figs. 3 and 4).

As another enhancement when analyzing SQL databases, the SQL statement that underpins the data element can now be viewed by the click of a button (see Fig. 4) instead of providing them only in the appendix section of the PDF report. These SQL statements can thus be copied from the GUI and pasted into a database tool to serve as a starting point for a more detailed investigation of possible irregularities identified by the DQA tool.

The *Plausibility Checks* are also visualized in a separate tab and were organized similarly to the *Descriptive Results*-Tab. Again, those checks are listed in sequential order in the PDF report. Now, the results for each plausibility check are displayed side by side to easily compare the results of the two databases under consideration. A sub-menu allows the user to select between the two implemented subcategories of the plausibility checks (*Atemporal Plausibility* checks and *Uniqueness Plausibility* checks), which are displayed as distinct screens (see Additional file 1: Fig. S5).

The *Completeness Check* screen presents a tabular summary of absolute and relative counts of missing values per data element to the user. Although these counts of missing values for the source and target database can be examined and compared, this view does neither offer an automated comparison of the two databases nor highlights notable attributes. Instead, the automated evaluation of the comparison of the absolute counts of missing values between a source and a target data set is presented on the main dashboard screen along with the “Completeness Checks (Validation)” (see right column “Check Missings” in Additional file 1: Fig. S3).

Besides the interactive presentation of the results in the GUI, the former PDF report can still be downloaded from the *Reporting* Tab (see Additional file 1: Fig. S6). A list of all database IDs associated with “conspicuous” values, for example that violate the value conformance or plausibility checks, and a summary of the check results presented on the dashboard, can also be downloaded as CSV files here. This information can be used to track and follow up on detected DQ irregularities directly in the databases.

For parametrizing the DQA tool, a screen was designed for setting default values during the GUI deployment, which is helpful when setting up the GUI for long-term use within a fixed infrastructure environment. Users can now select the desired databases to be tested (the information of available systems is taken from the MDR) on a new *Config* page. When provided during the initial deployment of the tool, predefined connection parameters for various databases are automatically inserted into the respective fields (see Additional file 1: Fig. S7), allowing users to connect to databases without technical knowledge. When all required parameters have been defined properly, a button is enabled from which the analysis can be triggered directly from this Config-page.

Finally, the *Logfile* tab displays all internal messages created during analysis and provides a full breakdown of the completed program steps (see Additional file 1: Fig. S8). During the iterative development of the interface, this was also a helpful source of information for troubleshooting software faults reported in the user feedback.

### Runtime

One user feedback from the first evaluation round addressed the rather long runtimes of the DQA tool when analyzing large data sets. Three enhancements, outlined in detail in the following section, addressed this aspect.

#### Selecting data elements

When utilizing DQAstats to perform a DQ analysis, the full set of data elements defined in the MDR for one database, or the intersection of data elements provided for two databases, will be examined. Sometimes, however, it is necessary to test only certain elements, e.g., newly added data elements only. The configuration page of the GUI was appended with the option of selecting the desired data elements for a DQ analysis to address this scenario (see Additional file 1: Fig. S7). This alteration restricts the analysis to data elements of interest and, reduces the tool’s overall runtime.

#### Time constraint for testing real-time data sets

During the initial phase of the MIRACUM project, most extract, transform, and load (ETL) jobs extracted the data from a clinical source system and transferred it to a research database in one batch. Therefore, all data was processed at once to make large data sets quickly available for analysis in the MIRACUM research data repositories. The MII-wide harmonization process to create a core data set and accompanying MII FHIR profiles was still in its early stages. As these processes advanced and since clinical routine data is rather dynamic and grows over time, the data-processing infrastructure was re-designed and adapted to take these developments into account. As a result, the former batch ETL jobs were re-implemented using Apache Kafka [29, 30] in order to support incremental data streams but at the same time ensuring compatibility with the MII FHIR profiles. ETL reimplementation makes the research infrastructure scalable, allowing it to manage real-time data generated in clinical practice and provide researchers with the most up-to-date information available. To allow meaningful DQ checks to be carried out on continuously growing databases, a new feature was added to the DQA tool for analyzing subsets of the databases based on time frames. Thus, the DQA tool is capable of examining subsets of research data repositories that are being filled in real time, as well as evaluating their filling ETL processes. As a side effect, selecting a smaller time frame also reduces the runtime.

#### Performance optimization

In the first version of the DQA tool, all DQ checks were processed sequentially for each data element. Suitable parts of the code were parallelized [31] to make the best use of available computing capacity while reducing analysis time and speeding up processing.

### Datamap

To centrally collect and report aggregated counts of selected data elements, a so-called *datamap* feature has been added.

**Fig. 5:**
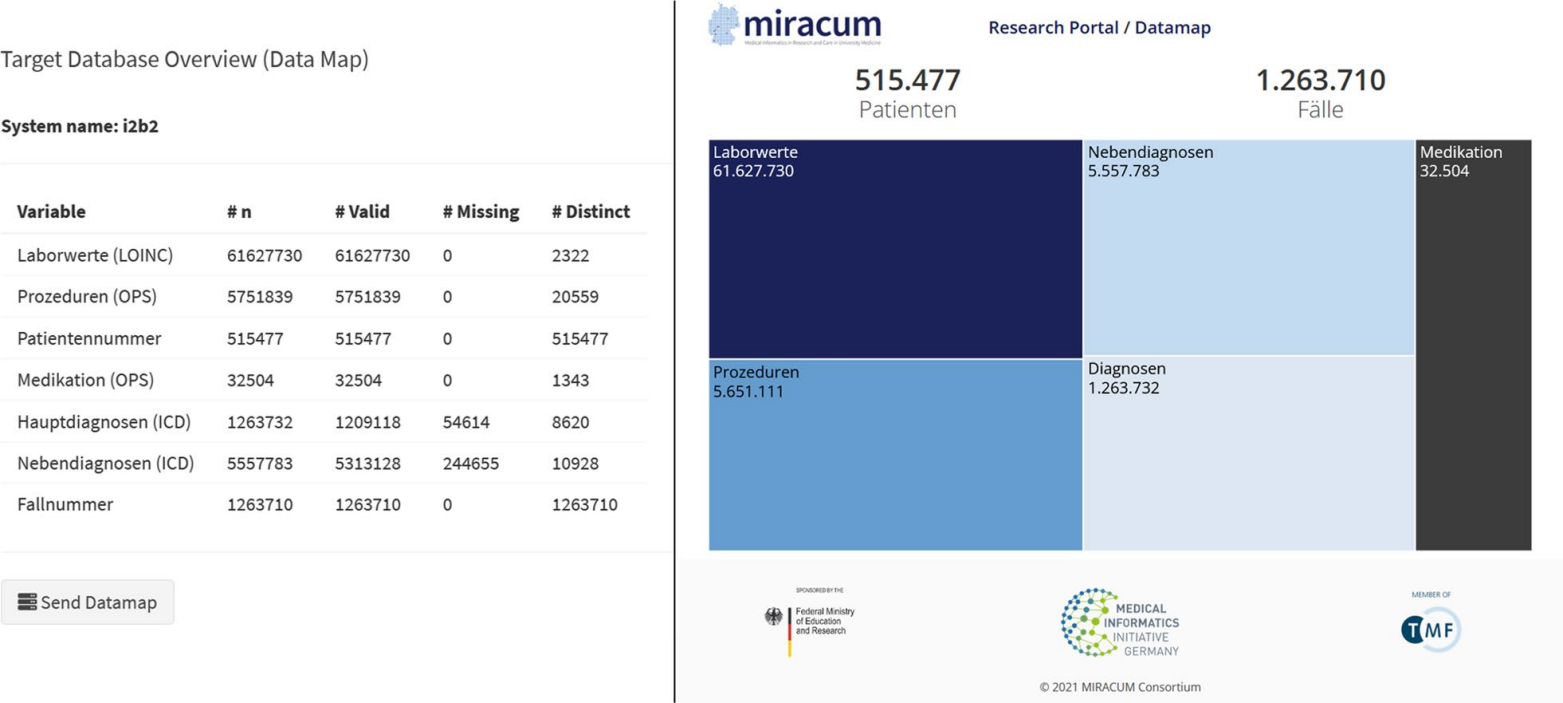
The MIRACUM datamap. Important data elements are displayed in a dedicated overview (datamap). On the left is an overview of the GUI elements which can be reviewed and sent to the datamap by clicking the button. On the right is a visualization of the availability of various data elements across all MIRACUM sites (see https://datamap.miracum.org/)

If an item has been marked in the MDR for inclusion in the datamap, it will be displayed prominently on the dashboard after a DQ analysis. When the analysis is complete, the datamap can be sent to a predetermined recipient by the partner sites by clicking a button. The datamap functionality within the MIRACUM project was prototypically extended to send aggregated counts to a central database. The counts provide a project-wide visualization of the availability of various data elements across all sites that is publicly available [32] (see Fig. 5). The datamap is intended to give researchers an initial overview of the quantity of selected data elements available at all sites before requesting the data to investigate their research questions.

### User feedback evaluation

Each release of the DQA tool was deployed at all ten MIRACUM sites. The first feedback round (FR1) finished at the end of 2019. During the process of this assessment, a total of 36 unique issues were reported. Subsequently, issues were allocated to one or more of four classifications: Eighteen issues were allocated to the class *LOGIC* (Problems that are caused by semantic or syntactic errors in the programming code), 3 issues to the class *ETL* (Problems that are caused by discrepancies in the ETL processes that populate the systems under test, rather than by faults in the DQA tool), 6 issues to the class *MDR* (Problems caused by inconsistencies in the metadata of the analyzed data elements, which, for example, led to incorrectly permitted value ranges), and 9 issues to the class *GUI* (Feedback on the graphical user interface of the DQA tool). Any reported issue could be allocated to multiple classes.

Furthermore, the feedback was prioritized based on its urgency and relevancy (see Additional file 1: section “Feedback round 1 (FR1)”). The most critical concerns were implemented by April 2020 and made available to all sites in an interim release. Additional issues were continuously addressed and provided to the sites in mid-2021 followed by another project-wide feedback round (FR2).

### Container-based application with Kubernetes support in MIRACUM

For providing the GUI-version of the DQA tool to the MIRACUM partner sites and to allow for a seamless integration into their local DIC, Docker was used to simplify the deployment of the application across different environments [33]. A container image [34, 35] was developed that integrates well within the MIRACUM DIC infrastructure, similar to the deployment of the command-line-based version of the DQA tool [20]. Since some sites already use Kubernetes for container orchestration, a Kubernetes manifest [35, 36] was also provided. The manifest leverages Argo workflows [37], an open source container-native workflow engine for orchestrating jobs on Kubernetes, to run automated DQ checks regularly. As a feature, this also updated the MIRACUM datamap with the latest metrics during its prototype development. Furthermore, practical aspects such as container availability (scheduling, scaling, and inter container communication) were addressed, enabling container orchestration in real time [33, 35, 36].

### MIRACUM enhancements and customizations

DQAgui was developed as a generic GUI-frontend built on top of DQAstats. Users without prior R programming knowledge can use it to analyze databases regarding their data quality and to compare different data sets. For customizing the DQA tool to the specific requirements within MIRACUM, such as connecting it to the central M-MDR and to provide the configurations to the MIRACUM research data repositories, the R package miRacumDQA was previously developed [20]. During the development of the GUI, this package was extended to also establish the connection to the prototype of the MIRACUM datamap.

Like DQAstats, the GUI was designed in a generic manner in order to be used for DQ checks independent of the project or data context. To demonstrate its applicability, the DQA tool includes synthetic data sets. A demo-instance is publicly available [38]. In the context of this paper and within the MIRACUM project, health data were analyzed.

## Discussion

With DQAgui a novel graphical user interface was developed that allows extensive data quality analyses to be performed by users without prior programming skills. In a research network, such as MIRACUM, this software can be used to check the DQ of large data sets at different sites in a standardized manner. The GUI frontend to the DQA tool was developed and distributed to all ten university hospitals within the German MIRACUM project. On the one hand, uniform checks can be provided via a central metadata repository to all partner sites to check, e.g., the compliance to a harmonized data model for several databases. On the other hand, the completeness of the data transferred to the research data repositories can also be checked by comparing it with the local databases at each site. In response to the user-centered development process, additional features were implemented such as the ability to time limit the analyzed data, and to restrict the analysis to specific data elements. Opposed to other solutions, like the data quality assessment tools for the OMOP CDM [39, 40], the DQA tool can be used for DQ checks of any (tabular) database, as long as the metadata is defined properly and correct SQL statements are provided for each data element. The interface focuses on a clear and structured visualization of DQ results, including a dashboard and a datamap with summary measures. The DQA tool was designed to be easy to use for DQ analysis of one or two data sets. In the MIRACUM research setting, the DQA tool was applied to assess DQ in longitudinal healthcare research data repositories.

Using the R package DQAstats, it is possible to perform DQ checks for two databases specified during the program call in the R console. As a result, a PDF report is created, that describes the results of the DQ checks in detail and sequential order for both analyzed data sets. With the R package DQAgui, the technical configuration of the tool was abstracted from the command line into a graphical user interface. The limitations of the previous PDF report, where DQ findings can be overlooked, were also addressed by an automated comparisons of the results of two different databases and their representation in the GUI, highlighting irregularities in color. As a result, users without prior programming skills are now able to perform DQ checks on large data sets.

Similar to the PDF report from DQAstats, the presentation of the DQ results in the GUI is again aligned to the harmonized DQA terminology from Kahn et al. [7].

However, one could argue that a potential disadvantage of using a GUI could be the lack of reproducibility of analysis workflows. By developing DQAgui as a frontend application on top of the logic implemented in DQAstats, the necessary features to conduct reproducible analyses are still available simply by parameterizing DQAstats. As a result, almost any of the analysis initiated through the GUI can also be triggered with DQAstats. Within the deployed version of the MIRACUM project’s GUI DQA tool, this feature is utilized to run automated DQ checks regularly using Argo workflows. The only restriction to DQAgui is that currently DQAstats is not able to pre-select specific data elements to be analyzed but only performs DQ checks for all data elements that are defined in the MDR.

The code of DQAgui is available under open source license on GitHub [41]. With the choice of R as a common open source programming language and the development of the whole DQA tool framework as R packages and Docker images, further enhancements to the software framework could easily be added in the future, similar to the extension of the framework with a GUI frontend and the adaptions for the usage within MIRACUM presented here.

The GUI was developed in an agile manner by quickly providing a minimum viable product to the end users and continuously improving it over time. The results of the iterative user-centered feedback rounds were very helpful for designing the interface and generally enhancing the functionality of the DQA tool. Issues were resolved early and the tool could be established MIRACUM-wide for local-site DQ review.

## Limitations

The development of the interface focused on creating a tool for DQ assessment that is intuitive and easy to use for users without prior programming knowledge. However, there are several limitations: First, while the development was primarily focused on making all functionality for DQ assessment from DQAstats available via a web-app based GUI, there were no distinct efforts regarding user-experience (UX) and, especially the design of the GUI. Instead, basic GUI elements such as checkboxes, buttons and lists, were chosen by common sense but without using specific criteria. Future efforts enhancing the tool could address this by specifically evaluating users workflow paths when performing DQ analyses, identifying possible GUI elements that could be exchanged by more appropriate ones to further simplify the DQ workflow.

Second, similar to DQAstats, DQAgui is designed to give a general view of the DQ of various databases and to validate ETL jobs that transfer data between two databases [42]. However, the tool neither assess DQ in terms of “fitness for use” [43], i.e. that the quality is sufficient to answer a specific research question, nor provides sophisticated statistical insights into the data, as dataquieR [10, 44] or the OMOP DQ tools do [39, 40]. Instead, our tools focus on providing a rapid assessment of the current status of DQ of one or two databases. This aspect is now better supported with the performance improvements and existence of the GUI as a web-app based frontend to DQ analyses from DQAstats, interactively presenting the results.

Furthermore the DQA tool does not currently cover all of the DQ sub-categories described by Kahn et al. [7]. However, based on the DQA tool’s output, it is possible to detect and fix observed discrepancies in the databases or ETL jobs that process the data.

In addition to the limitations mentioned above, which originate from the limitations of the DQAstats backend, there are also limitations solely related to the design of the GUI. R [25] was used because the MIRACUM project relies on a broad ecosystem of reusable open source tools for the architecture [12]. The open source language R, with its focus on statistical computing, fits this requirement. It was a natural choice to also develop the interactive GUI frontend in R using the R package shiny [26], although more appropriate programming languages and frameworks could exist therefore. This choice imposes, however, some limitations, such as a reduced flexibility when designing the GUI.

Finally, no standardized performance benchmarking was conducted, as the focus was on implementing important features and improving the interface. The amount of data to be analyzed, the DQ criteria to be checked, and the available computing capacity all contribute to the amount of time it takes to examine the DQ of a database. While the R console based version of the DQA tool provides status messages and technical details in the console during analysis, these technical details were deliberately placed on a separate tab of the user interface to keep the main screen clear (see Additional file 1: Fig. S8). As a result, users may get the impression that the tool is no longer responsive during the analysis, which can take a long time depending on the amount of data to be analyzed. A loading screen was added to counteract this issue and provide users with information about the status of the currently executed background activity [45]. Additional concepts, such as separating background tasks from the GUI, are conceivable here, for example, via the Argo Workflows API [37, 46–48].

### Outlook

A next step should include providing further visualizations and interactive evaluations of the examined data in the user interface. Additionaly, evaluating the usability of the DQA tool across all MIRACUM sites in a standardized and reproducible manner is also planned. Furthermore, developing a Helm Chart [49] to standardize and simplify the deployment within Kubernetes should be a next step.

## Conclusions

DQAgui is a novel graphical user interface to perform standardized data quality analyses as an extension to the MIRACUM DQA framework. Now equipped with a GUI, this software framework addresses a gap that was identified at the beginning of the MIRACUM project, when data quality checks were mainly performed inconsistently at the respective sites. The DQA tool enables users without prior programming knowledge both to carry out the DQ analyses and interpret the results directly in a web-browser. All features from the previously developed commandline-based R package DQAstats were integrated into the interface and new functions were added, including the ability to perform DQ analyses on subsets of the data by employing a time frame or by selecting the data elements to be analyzed. The tool was developed in a generic manner intended to be applicable to any tabular data set. In the context of this work, we demonstrate its application in the medical domain. Furthermore, the version of the DQA tool deployed within MIRACUM was linked to the publicly available centrally deployed datamap to summarize up-to-date information about the data available in the DIC across all MIRACUM sites.

## Supplementary Information

**Additional file 1.** Details about the user feedback received and available screens.

## Data Availability

The two R packages DQAstats and DQAgui are available on GitHub (https://github.com/miracum/dqa-dqastats, https://github.com/miracum/dqa-dqagui) and CRAN (https://cran.r-project.org/package=DQAstats, https://cran.r-project.org/package=DQAgui). Extensive information about the parameterization of the packages are available in the respective readme files, and a structured DQAstats wiki compilation (https://github.com/miracum/dqa-dqastats/wiki). The version of the DQA tool deployed within MIRACUM is available with the R package miRacumDQA (https://gitlab.miracum.org/miracum/dqa/miracumdqa). A demo version of the DQA tool is available online (https://dqa-demo.diz.uk-erlangen.de). The corresponding introduction for setting up the tool is included in the DQA tools Wiki (https://github.com/miracum/dqa-dqastats/wiki/DQAgui_intro).

## Abbreviations

BMBF: Federal Ministry of Education and Research in Germany (Bundesministerium für Bildung und Forschung)
DIC: Data integration center
DQ: Data quality
DQA: Data quality assessment
FR1: First feedback round
FR2: Second feedback round
GCP: Good clinical practice
GUI: Graphical user interface
EHR: Electronic health record
ETL: Extract transform load
MDR: Metadata repository
M-MDR: MIRACUM metadata repository
MIRACUM: Medical Informatics in Research and Care in University Medicine. German project which includes 10 German university hospitals
MII: Medical informatics initiative
UI: User interface
UX: User experience
