# Supplementary material for: DQAgui: a graphical user interface for the MIRACUM data quality assessment tool
Source: BMC Med Inform Decis Mak. 2022 Aug 11;22:213. doi: 10.1186/s12911-022-01961-z (PMC9367129; doi:10.1186/s12911-022-01961-z)
Supplement: Supplementary file 1 — Additional file 1. Details about the user feedback received and available screens. [file 12911_2022_1961_MOESM1_ESM.docx]

DQAgui: A Graphical User Interface for the MIRACUM Data Quality Assessment Tool

Additional file

Jonathan M. Mang^1,✉^, Susanne A. Seuchter^1^, Christian Gulden^2^, Stefanie Schild^1,2^, Detlef Kraska^1^, Hans-Ulrich Prokosch^1,2^, and Lorenz A. Kapsner^1,3^

12 June, 2022 - 19:02 UTC

^1^ Medical Center for Information and Communication Technology, Universitätsklinikum Erlangen, Erlangen, Germany.
^2^ Chair of Medical Informatics, Friedrich-Alexander-Universität Erlangen-Nürnberg (FAU), Erlangen, Germany.
^3^ Institute of Radiology, Universitätsklinikum Erlangen, Friedrich-Alexander-Universität Erlangen-Nürnberg, Erlangen, Germany.

^✉^ Correspondence: [Jonathan M. Mang <[jonathan.mang@uk-erlangen.de](mailto:jonathan.mang@uk-erlangen.de)>](mailto:jonathan.mang@uk-erlangen.de)

# Additional file 1

## Feedback round 1 (FR1)

Prioritizing the feedback is based on the severity ratings proposed by Nielsen [[1](#ref-nielsen1993)] and divided into 5 stages: Priority 0 (this is not a usability problem at all) was assigned to 2 problems, Priority 1 (cosmetic problem only — need not be fixed unless extra time is available on project) to 2 problems, Priority 2 (minor usability problem — fixing this should be given low priority) to 14 problems, Priority 3 (major usability problem — important to fix, so should be given high priority) to 17 problems, while there was no need to assign Priority 4 (usability catastrophe — imperative to fix this before product can be released) to any issue. Figure S1 depicts the results of the feedback round 1 (FR1). The most serious inaccuracies may be seen in categories LOGIC and MDR, where 11 of the 17 recorded responses (65%) were incorrectly identified as faults and were the result of misconceptions. One feedback addressed the missing values for the variable “admission age in days”, which affected the majority of observations at the local site. The relevance of this issue, however, is quite low because this information is only reported for patients of age of less than one year, as can be read in the official §21 data dictionary [[2](#ref-Gesetza)]. This feedback was categorized as “irrelevant” or “rejected” due to its contextual reference, the fact that they are unrelated to the DQA tool, or the fact that they mention the same concerns twice. The remaining 6 concerns with a priority greater than or equal to priority 3 were fixed and implemented in the subsequent version of the DQA tool provided to all sites in April 2020, with one exception that required a change of the MIRACUM MDR. As an example for an issue with priority higher than or equal to Priority 3, several users discovered that the total number of data records evaluated for a data element did not match the equivalent information in the PDF report, which could be identified as a bug in the code. This type of feedback was crucial for the validation and designing process of the interface.


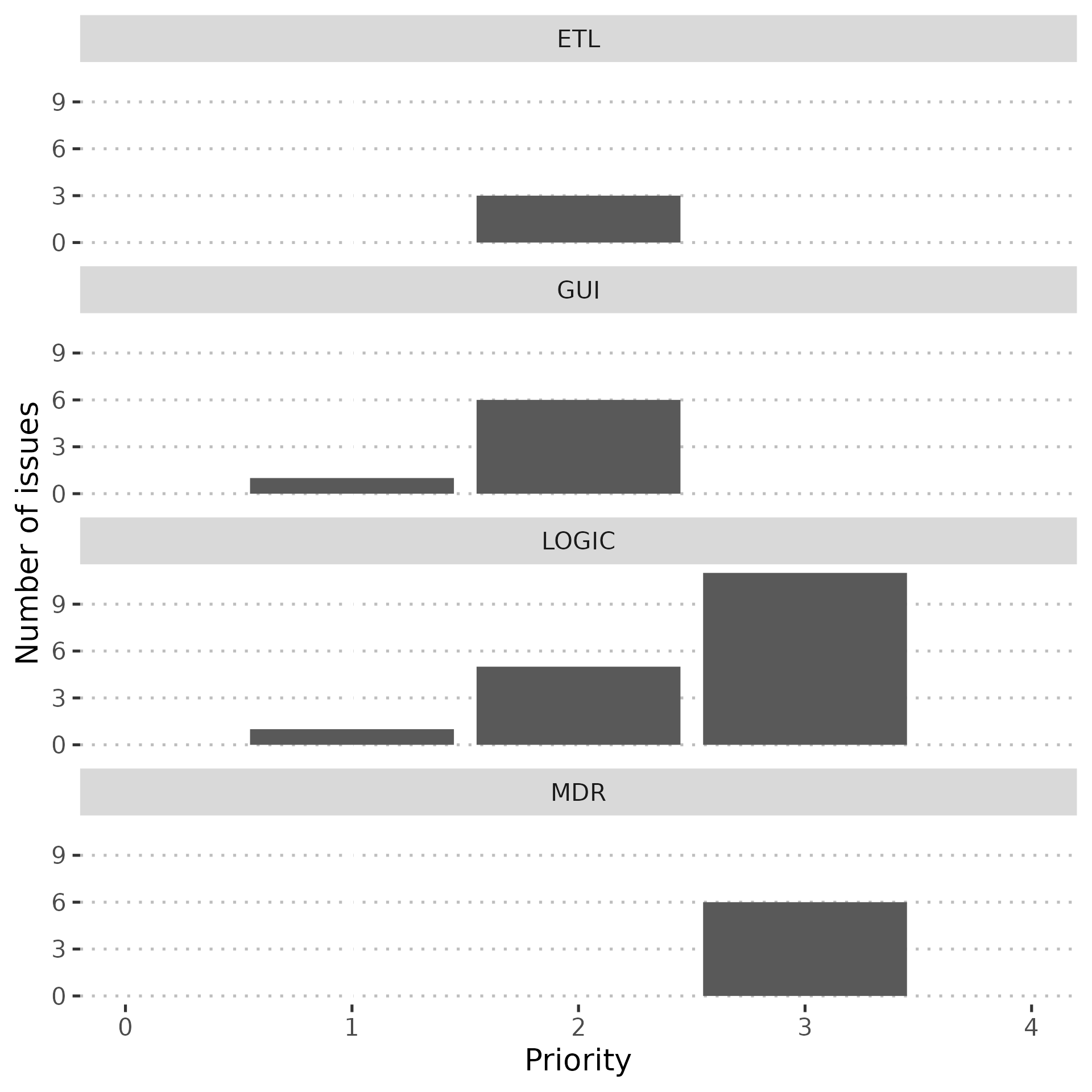


Figure S1: Classification of the feedback from feedback round 1 (FR1). In total, n=36 feedback responses were classified and prioritized after evaluating the DQA tool enhanced with the graphical interface across all 10 MIRACUM sites. Priority 0 = This is not a usability problem at all; Priority 4 = Usability catastrophe — imperative to fix this before product can be released.

## GUI screens


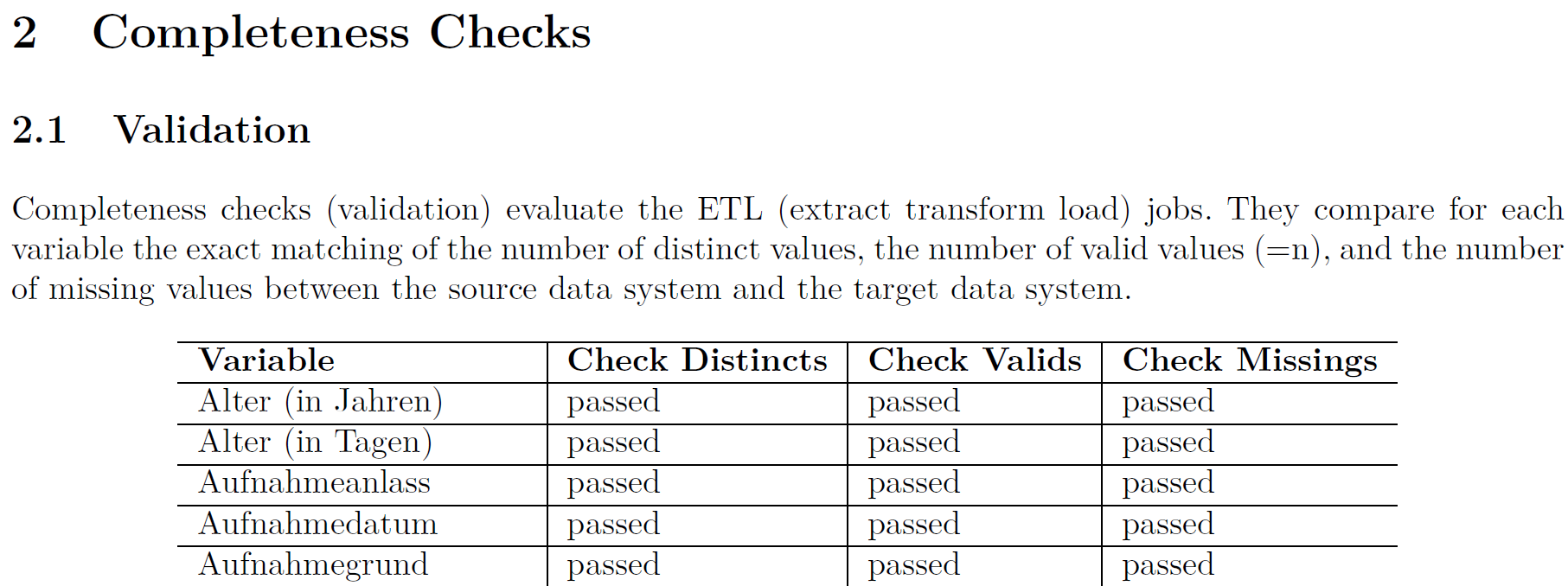


Figure S2: Representation of completeness checks in the PDF report.


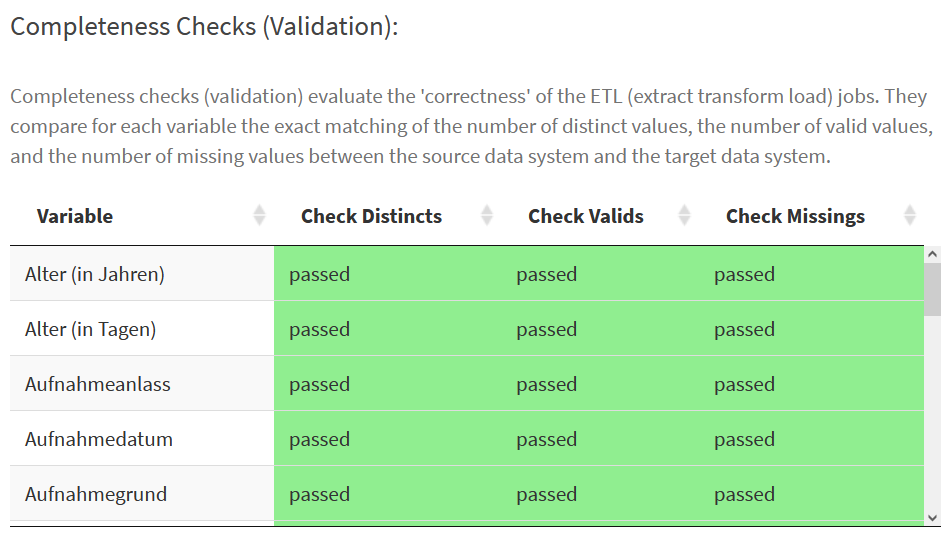


Figure S3: Representation of completeness checks in the GUI. The web-based interface displays highlighted results per data element.


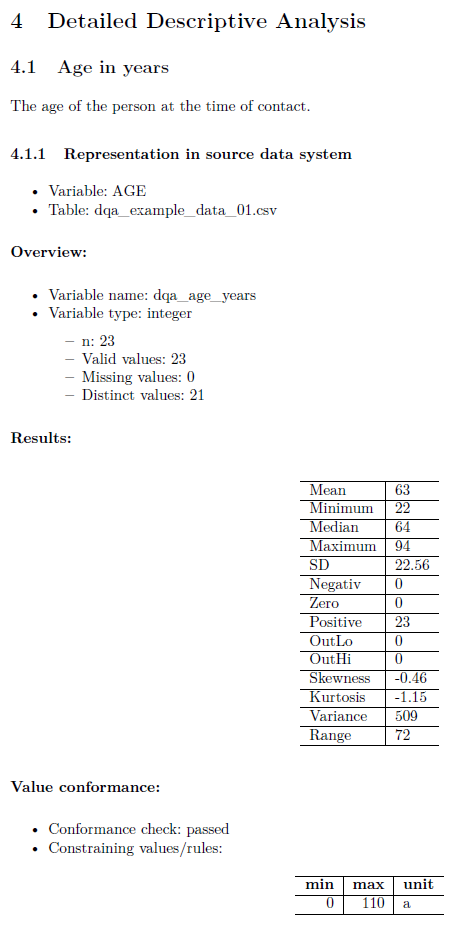


Figure S4: Representation of descriptive analysis results for a single data element in the PDF report.


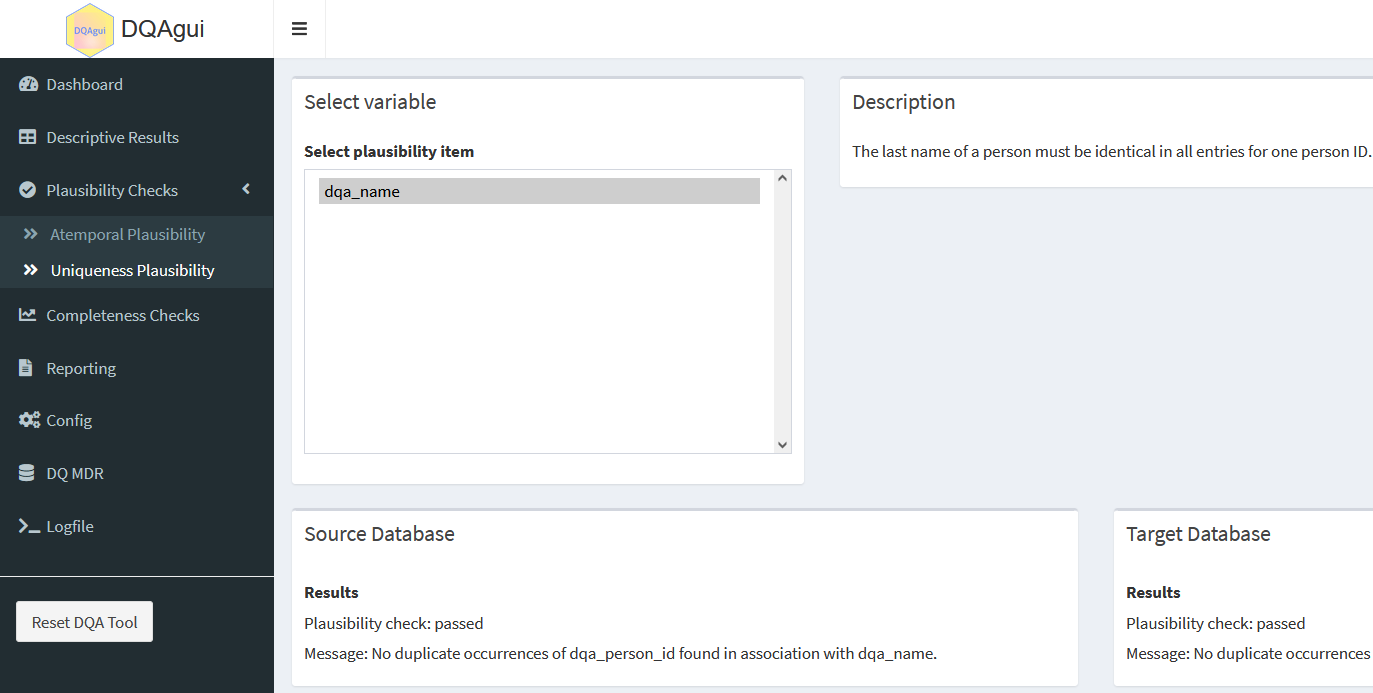


Figure S5: Menu structure of the GUI. The menu navigation on the left is aligned to data quality categories proposed by Kahn et al. (2016) [[3](#ref-kahn2016)] and allows the user to switch between the different DQ check results and the tools settings.


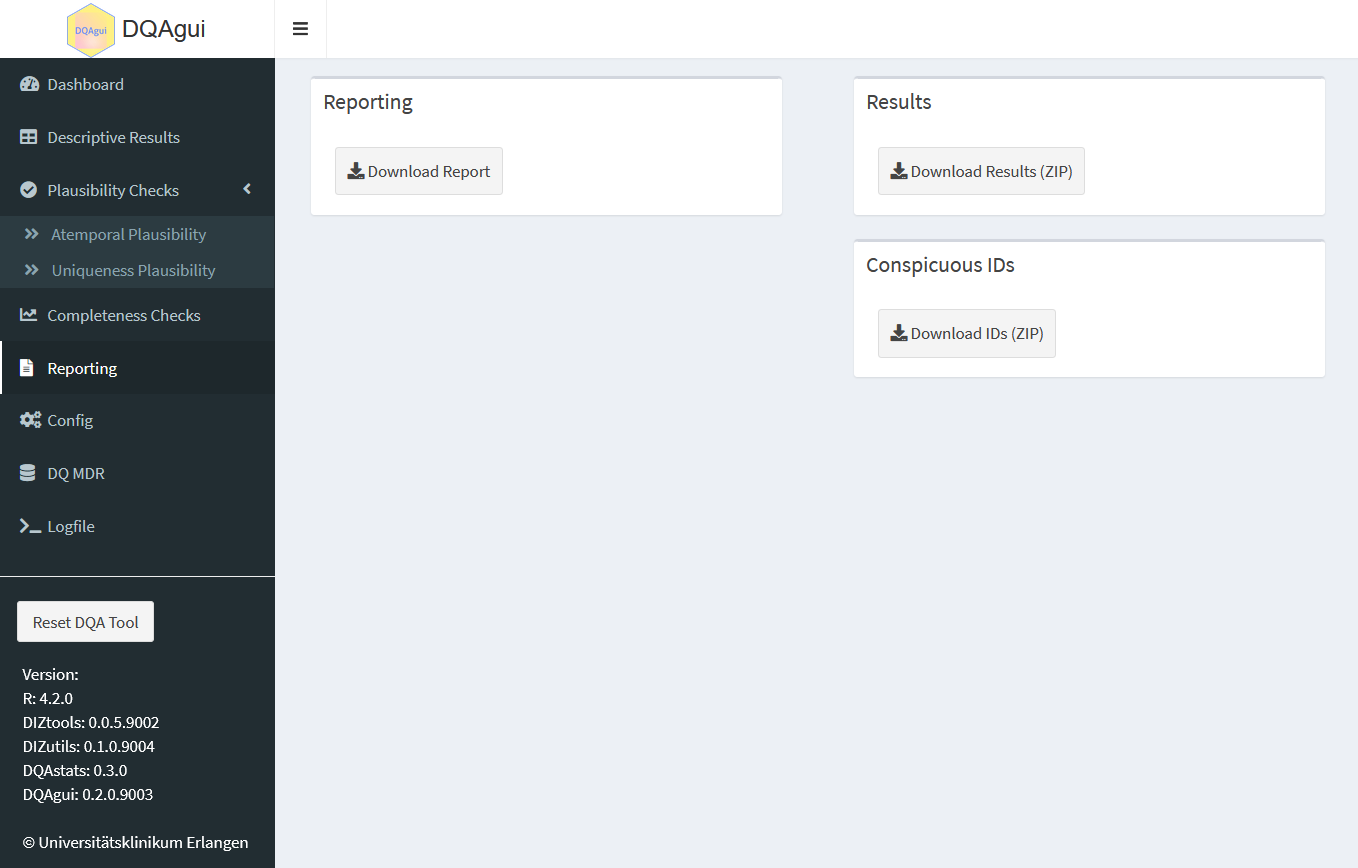


Figure S6: The reporting screen. Reporting tab with the possibility to download the PDF report as well as all results as tables including the conspicuous values found during the DQ checks.


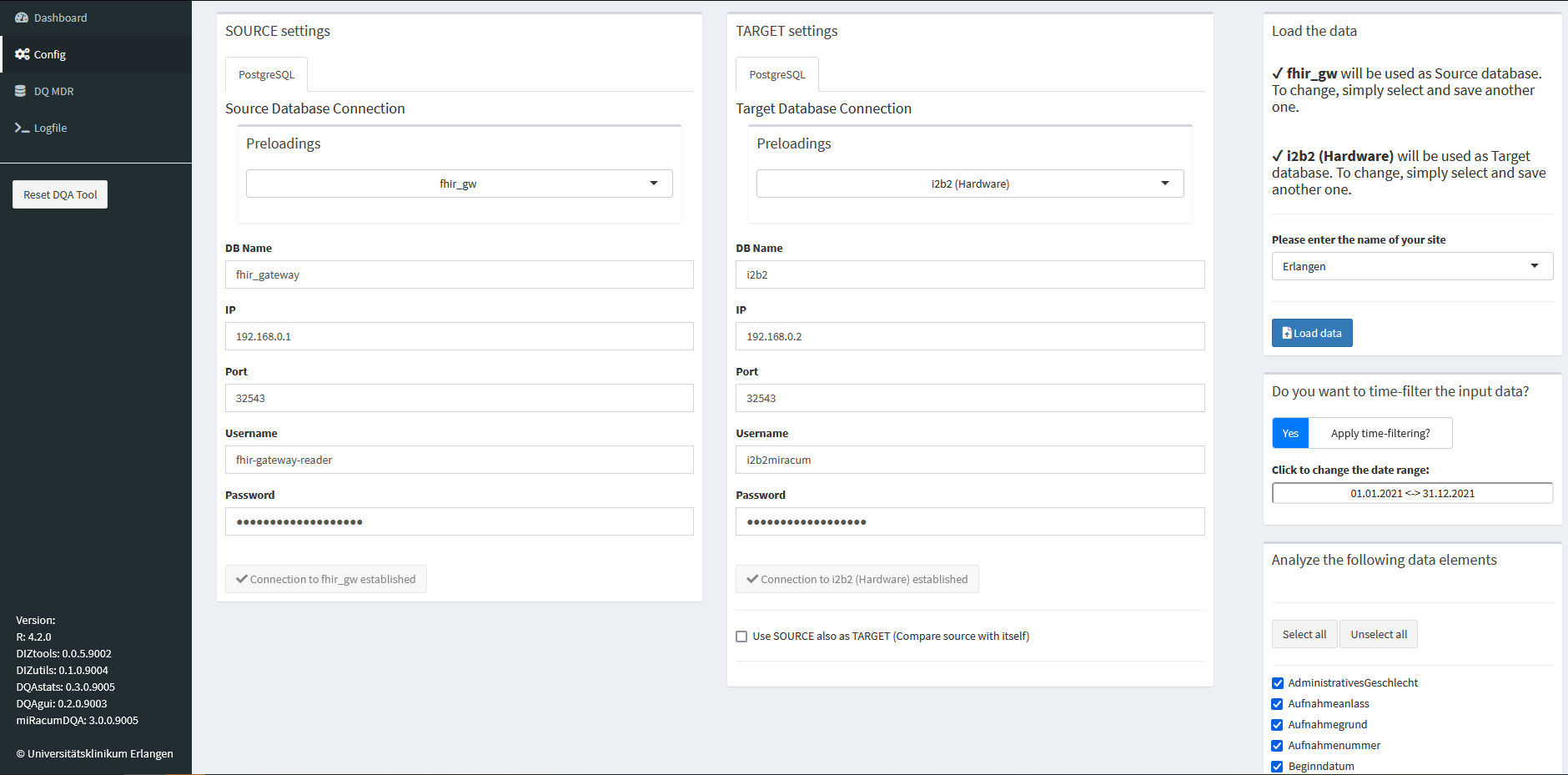


Figure S7: The configuration screen. The connection parameters, data elements to be examined, and the time range to be tested can be entered in the Config tab. The analysis can be triggered by clicking the “Load data” button, once all systems have been configured successfully.


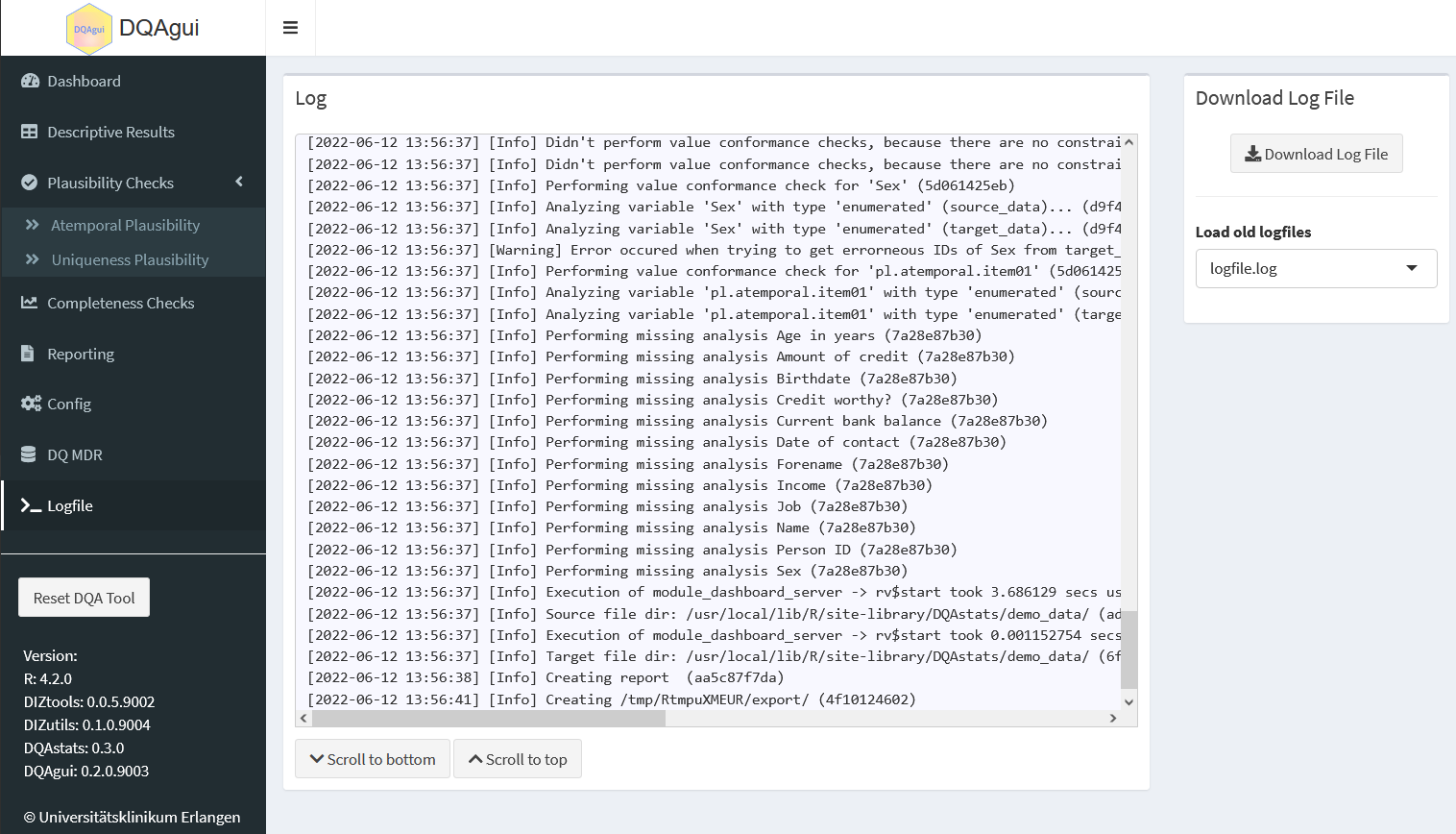


Figure S8: The logging screen. All steps performed by the DQA tool are protocolled within the Logfile tab. This was especially useful for cross-site troubleshooting.

# References

1. Nielsen J (1993) [Usability engineering](https://doi.org/10.1016/C2009-0-21512-1). Morgan Kaufmann Publishers In, San Francisco

2. [Gesetz über die Entgelte für voll- und teilstationäre Krankenhausleistungen (Krankenhausentgeltgesetz - KHEntgG) § 21 Übermittlung und Nutzung von Daten](https://www.gesetze-im-internet.de/khentgg/__21.html)

3. Kahn MG, Callahan TJ, Barnard J, et al (2016) A Harmonized Data Quality Assessment Terminology and Framework for the Secondary Use of Electronic Health Record Data. eGEMs (Generating Evidence & Methods to improve patient outcomes) 4:18. <https://doi.org/gf93nz>
